# Resistant Starch as a Functional Nutrient to Control Cardiometabolic Risk Factors in Humans: An Integrative Review

**DOI:** 10.1007/s13668-026-00766-0

**Published:** 2026-04-30

**Authors:** Isabela Ribeiro Grangeira Tavares, Eduardo Éric Almeida do Carmo, Thiago da Silveira Alvares

**Affiliations:** 1 Food Analysis and Nutritional Biochemistry Laboratory, Food and Nutrition Institute, Multidisciplinary Center UFRJ-Macaé, Macaé, RJ Brazil; 2 Food and Nutrition Institute, Multidisciplinary Center UFRJ-Macaé, Macaé, RJ Brazil

**Keywords:** Clinical nutrition, Functional carbohydrates, Energy metabolism, Non-communicable chronic diseases, Dietary intervention, Clinical trials

## Abstract

**Purpose of Review:**

Resistant starch (RS) has been widely investigated as a dietary component with potential metabolic benefits, including improved insulin sensitivity, lipid profile, and inflammatory markers. However, clinical findings remain inconsistent, particularly regarding RS type and dosage. This integrative review aimed to synthesize evidence on the effects of RS consumption in dietary interventions on metabolic and cardiovascular parameters in adults and older adults.

**Recent Findings:**

RS intake, particularly RS2 and RS3, was associated with significant reductions in postprandial glucose, insulin, and HOMA-IR, as well as improvements in total cholesterol, LDL-C, and triglycerides. Additional findings indicated modest decreases in blood pressure and central adiposity, linked to increased short-chain fatty acid production and higher GLP-1 and PYY levels.

**Summary:**

Despite promising results, methodological heterogeneity and short intervention durations limit the strength of conclusions. RS shows potential as a functional nutrient for cardiometabolic modulation, particularly for glycemic and lipid control. However, longer, standardized clinical trials are required to confirm efficacy and clarify its physiological mechanisms.

**Graphical Abstract:**

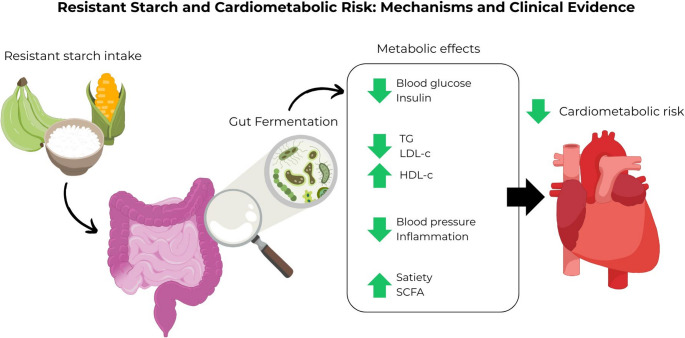

## Introduction

Cardiometabolic diseases such as type 2 diabetes mellitus (T2DM), hypertension, obesity, and dyslipidemias rank among the leading causes of morbidity and mortality worldwide, representing one of the most significant challenges to contemporary public health [1, 2]. Their increasing prevalence has been strongly associated with the presence of multiple cardiometabolic risk factors—including elevated systolic blood pressure, high low-density lipoprotein cholesterol (LDL-c), elevated body mass index (BMI), and elevated fasting glucose—often exacerbated by unhealthy lifestyle behaviors such as physical inactivity and poor dietary habits [3]. According to the World Health Organization (WHO), these risk factors are interrelated and play a key role in the development of non-communicable diseases (NCDs), particularly cardiovascular diseases. Among them, elevated systolic blood pressure stands out as the leading modifiable risk factor for premature cardiovascular deaths worldwide, accounting for more than 10 million deaths globally in 2021 [3, 4]. Therefore, understanding strategies that can modulate these cardiometabolic determinants is essential for reducing the burden of these diseases in adult and elderly populations.

Dietary interventions have proven effective in preventing and managing cardiometabolic risk factors through mechanisms that include improved insulin sensitivity and lipid metabolism [5]. Evidence suggests that balanced diets rich in dietary fiber and bioactive compounds are associated with better metabolic and inflammatory regulation [6]. In this context, resistant starch (RS) has gained prominence in the scientific literature as a functional component that can modulate blood glucose, insulin sensitivity, and the gut microbiota, potentially contributing to cardiometabolic health [7].

Resistant starch refers to the fraction of starch that resists digestion in the small intestine and reaches the colon, where it undergoes fermentation by the gut microbiota, producing short-chain fatty acids (SCFAs) that may exert metabolic benefits. RS has been associated with improvements in insulin sensitivity, glycemic control, and supporting gut health [8]. Based on its structural characteristics and resistance to enzymatic digestion, RS has been classified into five main types (RS1-RS5), which differ in their physical structure, sources, and physiological effects [9]. RS1 consists of physically inaccessible starch trapped within intact plant cell walls, typically found in whole or partially milled grains and seeds. RS2 refers to native granular starch with a compact crystalline structure that resists enzymatic digestion, present in foods such as raw potatoes, green bananas, and high-amylose maize. RS3 is a retrograded starch formed when gelatinized starch is cooled after cooking, allowing molecular reorganization that increases its resistance to digestion, and is commonly present in cooled potatoes, rice, and pasta. RS4 corresponds to chemically modified starches produced through industrial processes to enhance functional properties in food processing. Finally, RS5 consists of amylose–lipid complexes formed during food processing or artificially created resistant maltodextrins [8]. To facilitate understanding of these different forms, Table 1 summarizes the main types of resistant starch, their structural characteristics, common dietary sources, and the metabolic effects described in the literature. Among the different types of resistant starch described in Table 1, RS2 (native granular resistant starch) and RS3 (retrograded resistant starch) are the forms most frequently investigated in human clinical studies, particularly regarding their effects on glycemic control and cardiometabolic risk factors.

**Table 1:** Main types of resistant starch, dietary sources, and reported metabolic effects

RS Type	Structural Characteristics	Main Food Sources	Metabolic Effects Reported in the Literature
RS1	Physically inaccessible starch trapped within intact plant cell walls	Whole grains, vegetables, and seeds	Slower starch digestion reduces the glycemic response and may improve insulin sensitivity
RS2	Native granular starch is resistant to enzymatic digestion	Raw potatoes, green bananas, high-amylose maize starch	Improved insulin sensitivity, reduced postprandial glucose and insulin, increased SCFAs production
RS3	Retrograded starch formed after cooking and cooling starchy foods	Cooled rice, potatoes, pasta, bread	Improved glycemic control, enhanced satiety, and beneficial modulation of gut microbiota
RS4	Chemically modified starch produced through industrial processes	Modified starches are used in processed foods	Possible reduction in postprandial glucose and triglycerides; effects vary depending on modification type.
RS5	Amylose-lipid complexes formed during processing	Processed foods containing amylose-lipid interactions	Emerging evidence suggests reduced starch digestibility and potential metabolic benefits

Despite growing evidence supporting the metabolic benefits of RS [10], a comprehensive and critical synthesis of the literature is still needed to clarify its specific role in controlling cardiometabolic risk factors. Although individual studies have demonstrated the effects of RS on insulin sensitivity [11], postprandial glucose control [12], and lipid metabolism [13], substantial heterogeneity in the types of RS evaluated, large variability in intervention protocols – including differences in RS dose, duration of supplementation, and food matrices- complicates comparisons across studies. Furthermore, most trials involve relatively small sample sizes and short intervention periods, limiting the ability to draw firm conclusions about the long-term cardiometabolic effects of RS consumption. In addition, there is limited clinical evidence regarding RS1, RS4, and RS5, despite their potential physiological relevance. Finally, the interaction between resistant starch fermentation, gut microbiota composition, and cardiometabolic responses remains incompletely understood. For this reason, integrative analyses that synthesize current clinical evidence are necessary to clarify the role of RS in modulating cardiometabolic risk factors.

Therefore, this review aims to synthesize evidence from controlled clinical trials evaluating the effects of resistant starch consumption on cardiometabolic risk factors, including insulin resistance, blood pressure, lipid profiles, and body composition, in adults and older adults, and to identify consistent findings, methodological limitations, and remaining gaps in the literature. By synthesizing the results of clinical trials, this review seeks to provide a clearer understanding of RS’s potential as a functional nutrient for cardiometabolic health and to identify areas for future research in this field.

## Materials and Methods

### Search Strategy

An integrative literature review was conducted based on a systematic search in the PubMed, Scopus, and Web of Science databases, covering the period from January 2015 to October 2025. The descriptors used were “nutritional intervention” AND “resistant starch,” combined using Boolean operators to locate relevant studies. Only publications in English with an available abstract were considered.

Original studies were included if they evaluated dietary interventions containing resistant starch (RS), reported the amount used in the nutritional intervention, and assessed at least one of the following outcomes, including glycemic parameters (fasting glucose, insulin, homeostatic model assessment for insulin resistance [HOMA-IR]), lipid profile (total cholesterol, high-density lipoprotein cholesterol [HDL-c], LDL-c, triglycerides), blood pressure, or body composition.

**Fig. 1:**
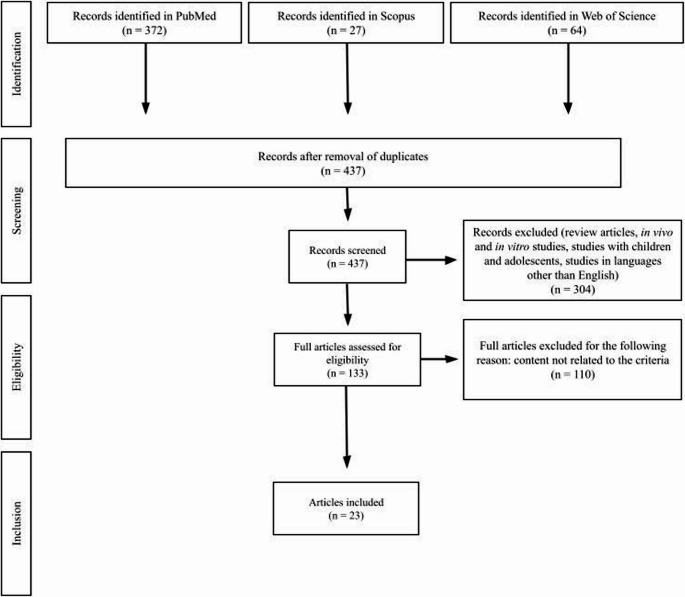
Flow diagram of the literature search and selection process

Exclusion criteria included review articles, meta-analyses, study protocols, case reports, publications without full-text access or without an available abstract, studies conducted in children or adolescents, and investigations performed exclusively in vitro or in animal models, and duplicate articles across databases. The process of identifying, screening, assessing eligibility, and including studies is illustrated in Fig. 1.

The database search returned 460 records. After duplicate removal and title and abstract screening, 133 articles were assessed in full text. Of these, 23 studies met all eligibility criteria and were included in the final synthesis.

### Eligibility Criteria

Randomized clinical trials and controlled clinical trials were included if they compared cardiometabolic risk factors before and after RS intervention. Only studies conducted with adults (≥ 18 years), including older adults of both sexes, with or without a prior diagnosis of cardiometabolic conditions, were eligible.

Studies were required to assess at least one primary cardiometabolic outcome, such as insulin resistance, lipid profile, blood pressure, or body composition. Studies that analyzed secondary effects related to these outcomes were also accepted, provided that RS was the main component of the dietary intervention.

### Study Selection

Screening was conducted in two stages. First, titles and abstracts were independently reviewed by two researchers to exclude articles that were clearly irrelevant. Then, the full texts of potentially eligible studies were reviewed to verify adherence to inclusion and exclusion criteria. Disagreements between reviewers were resolved through discussion and consensus. If consensus was not reached, a third reviewer was consulted to make the final decision, ensuring greater reliability in the selection process.

### Data Extraction and Synthesis

Data extraction from the included studies was performed in a standardized manner using a pre-designed spreadsheet. Information collected included: authors and year of publication, sample characteristics, type and source of resistant starch used, dose and duration of the intervention, outcomes evaluated, and main results and conclusions.

Data synthesis was conducted descriptively and comparatively, considering similarities and differences across studies in terms of RS type, sample characteristics, intervention duration, and the magnitude of observed effects.

Overall, 23 studies met the inclusion criteria and were analyzed in this review. Among these, the majority reported beneficial metabolic effects, including reductions in postprandial glucose and insulin levels, improvements in HOMA-IR, and favorable changes in lipid parameters, including total cholesterol, LDL-C, and triglycerides. However, fewer studies reported neutral effects, particularly in shorter interventions or in metabolically healthy populations, and few reported no significant metabolic changes. These variations highlight the importance of considering factors such as RS type, dose, intervention duration, and participants’ baseline metabolic characteristics.

## Results

### Study Selection Process

The systematic search in the PubMed, Scopus, and Web of Science databases yielded a total of 460 records. After removing duplicates and screening titles and abstracts, 133 articles were assessed in full text, of which 23 met the inclusion criteria. The eligibility process is illustrated in Fig. 1.

The final sample includes both acute studies (postprandial/test-meal designs) and chronic trials (mostly 4–12 weeks), conducted in diverse populations: healthy adults, individuals with overweight/obesity, prediabetes/type 2 diabetes, nonalcoholic fatty liver disease (NAFLD), and hyperlipidemia. The main methodological characteristics and results of the studies are summarized in Table 2.

**Table 2:** Potential health benefits of RS interventions on cardiometabolic risk factors

Reference	Sample / Study design	Intervention	Results
Ren et al. (2024) [38]	56 patients with hyperlipidemia (32 men and 24 women) Randomized, controlled	Polished rice (PR) or AGBR (2.85 g/100 g of RS) for lunch (200 g) and dinner (120 g) for 3 months.	↓ TG, TC, and LDL-c ↑ HDL-c
Lúcio et al. (2023) [28]	21 overweight men Randomized, controlled, single-blind	40 g of extruded sorghum SC319 or 38 g of extruded whole wheat with a calorie restriction of 500 kcal/day for 8 weeks.	↓ BW, WC, SAD, WR, %BF
Ni et al. (2023) [24]	200 adults with NAFLD (145 men and 55 women) Randomized, double-blind, parallel	40 g/day of type 2 resistant starch derived from high-amylose corn (HAM-RS2) or consumption of control starch with equivalent energy value for 4 months.	↓ FG, FI, HOMA-IR, TC, TG, LDL-c, SBP, DBP, BW, WC, HC, WR, BF, FM, VFI, LM ↑ HDL-c
Astina et al. (2022) [29]	22 adults with prediabetes and normoglycemic (9 men and 13 women) Single-arm prospective	56 g of TRM30 (30% TRM substitution) for 12 weeks.	↔ BW, FM, MM, FBG, FI, TC, LDL-c, HDL-c, TG ↑ VFI prediabetic group ↓ HbA1c
Hughes et al. (2021) [16]	30 healthy subjects with a body mass index (BMI) of 26.5 ± 3.8 Kg/m² (12 men and 18 women) Randomized, double-blind, crossover, and placebo-controlled.	Bread enriched with RS2 (14–19 g of RS/day) or conventional wheat (2–3 g of RS/day) for 1 week.	↓ iAUC of glucose, iAUC of insulin, Glucose peak, Insulin peak
Johnstone et al. (2020) [26]	19 overweight adults (11 men and 8 women) Randomized, crossover, and placebo-controlled	Maintenance diet for 3 days, followed by a weight loss diet for 21 days, and then 2 maintenance diets of 10 days each, containing RS type 3 (RS-WM − 22 g/day for women and 26 g/day for men) or no RS (C-WM).	↔ BW ↓ WC, HC, TC, LDL-c, SBP, DBP, HR after the WL diet ↓ FG after the RS-WM diet ↔ FI levels between the RS-WM and C-WM diets ↓ TG after the C-WM and RS-WM diet periods
Costa et al. (2019) [14]	131 individuals with prediabetes and type 2 diabetes Prospective, randomized, open-label trial, parallel arms, and blinded endpoints	Dietary intervention plus green banana biomass (G1–40 g of green banana biomass daily [4.5 g of RS]) or the dietary intervention alone (G2) for 24 weeks.	↓ FG, HbA1c, BW, BMI, DBP in the G1 group ↓ HDL-cholesterol in the G2 group ↓ WC, HC in the G1 and G2 groups
Meng et al. (2019) [25]	75 patients with early-stage type 2 diabetic nephropathy (34 men and 41 women) Randomized, comparative, open-label, parallel	50 g/day (~ 17.41 g/day of resistant starch) of flour rich in resistant starch and low in protein, twice a day (lunch and dinner) for 12 weeks.	↓ FG, HbA1c, TC, TG
Zhang et al. (2019) [17]	19 healthy adults Randomized, crossover, double-blind	40 g/day of type 2 resistant starch from high-amylose corn or regular starch with equivalent energy value for 4 weeks.	↓ LDL-c, VFI, SAD ↑ FI, C-peptide ↔FG, TG, BW, WR, BF, FM, MM
Peterson et al. (2018) [22]	59 prediabetic subjects (20 men and 39 women) Randomized, double-blind, placebo-controlled	45 g/day of type 2 resistant starch (RS2) from high-amylose corn (HAM-RS2) or an isocaloric amount of rapidly digestible corn starch (control) for 12 weeks.	↔ BW and FM, Glucose and insulin, IVGTT, SBP, Lipid profile ↔ Visceral fat, IMCL in the tibialis anterior muscle, HbA1c in the RS2 group ↓ EMCL in the soleus muscle, HR in the RS2 group
Stewart and Zimmer (2018) [27]	28 healthy adults (14 men and 14 women) Randomized, double-blind, controlled crossover	Muffin with 11,6 g of fiber from type 4 resistant starch (RS4 - VERSAFIBE™ 2470) or muffin with 0,9 g of fiber, equal in weight, total carbohydrates, protein, and fat	↓ FG, FI
Stewart et al. (2018) [19]	36 healthy adults with a BMI of 26.1 ± 0.5 kg/m² (12 men and 23 women) Double-blind, randomized, controlled	High-fiber scone containing RS4 (VERSAFIBE™ 2470) or a low-fiber control scone without RS4.	↓ iAUC for venous and capillary glucose, iAUC for venous insulin, Cmax of venous and capillary glucose, venous insulin
Emilien et al. (2017) [20]	27 healthy adults (15 men and 12 women) Randomized, single-blind, crossover clinical trial	Muffins with 40% of wheat flour replaced by resistant wheat starch (Fibersym^®^), totaling 26 g of fiber per meal, or muffins with standard wheat flour, containing only 2 g of fiber	↓ FI ↔FG
Maziarz et al. (2017) [32]	18 healthy overweight adults (3 men and 15 women) Double-blind, randomized-controlled, parallel-arm trial	Muffins enriched with 30 g of HAM-RS2 or control muffins were given daily for 6 weeks.	↓ AUC of postprandial glucose in the HAM-RS2 group ↔ Insulin, BM, FM, LM, and visceral fat mass
Rahat-Rozenbloom et al. (2017) [23]	25 healthy overweight/obese and lean participants (12 men and 13 women) Crossover, randomized, controlled	300 mL of water containing 75 g glucose (GLU) as a control, or 75 g GLU plus 24 g IN, or 75 g GLU plus 28.2 g RS.	↑ iAUC of glucose 0–2 h after consumption IN vs. GLU ↓ tAUC 4–6 h after consumption of RS vs. GLU, tAUC of insulin 4 to 6 h after consumption of RS vs. GLU ↔ iAUC of insulin from 0–2 h ↑ iAUC of insulin from 2 to 4 h after consumption of RS vs. GLU
Stewart and Zimmer (2017) [18]	28 healthy adults (14 men and 14 women) Randomized, double-blind, controlled, crossover	A cookie with 24 g fiber from type 4 resistant starch (RS4 - VERSAFIBE™ 1490) or a cookie with 0.5 g of fiber, equal in weight, total carbohydrates, proteins, and fats	↓ FG, FI
Dainty et al. (2016) [15]	24 adults at risk of T2D (16 men and 8 women) Crossover, randomized, double-blind	A bagel containing 25 g of HAM-RS2 or a control bagel daily for 56 days	↔ FG, iAUC of postprandial glucose ↓ FI, iAUC of postprandial insulin, HOMA-IR, HOMA-%B ↑ HOMA-%S
Mohan et al. (2016) [34]	30 healthy adults (subsample of 15 participants) Randomized, controlled, crossover clinical trial	High-fiber white rice (3.9 g/100 g of resistant starch), developed by conventional genetic improvement, or commercial white rice	↓ FG
O’Connor and Campbell (2016) [33]	20 healthy young adults Randomized, double-blind, crossover clinical trial	Drink or bar containing a fiber compound with high amylose corn starch (RS2) + guar gum or maltodextrin product, equal in calories and macronutrients	↓ FG, FI
Gargari et al. (2015) [21]	60 women with type 2 diabetes Controlled, randomized, triple-blind	10 g/day of RS2 (Hi-maize 260) or 10 g/day of maltodextrin as placebo, for 8 weeks	↓ FPG, HbA1c, TG ↔ TC, LDL-c ↑ HDL-c
Gentile et al. (2015) [31]	16 lean and overweight/obese women Crossover, randomized, single-blinded	One of four test meals: (1) waxy corn starch (control) (WMS); (2) waxy corn starch with whey protein (WMS + WP); (3) resistant starch (RS); or (4) resistant starch with whey protein (RS + WP).	↔ Postprandial glucose, Postprandial insulin ↓ % Fat oxidation TEM RS + WP
Nilsson et al. (2015) [35]	20 healthy middle-aged adults Randomized crossover	Barley grain bread, rich in fiber and resistant starch (17 g/day), or white wheat bread for 3 days	↓ FGB, FI ↑ ISI
Sonia et al. (2015) [30]	15 healthy adults (5 men and 10 women) Randomized, crossover, single-blind	Cooked white rice, cooled for 24 h at 4 °C and reheated (RS = 1.65 g/100 g) or freshly cooked white rice (RS = 0.64 g/100 g)	↓ FGB

*TG* Triglycerides, *TC *Total Cholesterol, *HDL-c* High-Density Lipoprotein cholesterol, *LDL-c* Low-Density Lipoprotein cholesterol, *BW* Body Weight, *WC* Waist Circumference, *SAD* Sagittal Abdominal Diameter, *WR* Waist-to-height Ratio, *BF* Body Fat, *FM* Fat Mass, *MM* Muscle Mass, *FGB* Fasting Blood Glucose, *FI* Fasting Insulin, *HC* Hip Circumference, *HR* Heart Rate, *VFI* Visceral Fat Index, *LM* Lean Mass, *FG* Fasting Glucose, *BM* Body Mass, *ISI* Insulin Sensitivity Index, *FPG* Fasting Plasma Glucose

### General Characteristics of the Interventions

The types of RS tested in the included studies were primarily RS2 (high-amylose corn starch, also known as Hi-Maize^®^), RS3 (retrograded starch from cooled foods/grain treatments), and RS4 (chemically modified starches). Delivery formats ranged from isolated supplements (e.g., Hi-Maize^®^) to functional foods (breads, cookies, muffins, treated rice, flours). Reported doses ranged widely: acute studies used portions containing 10 to 40 g of RS per meal; chronic trials reported approximately 10 to 45 g/day, with more consistent effects observed from 15 g/day onward. Intervention durations ranged from single acute tests to chronic periods of 4 to 12 weeks.

### Effects on Glycemic Metabolism and Insulin Sensitivity

Most trials reported improvements in glycemic control and insulin sensitivity, especially with RS2 and RS3. Studies in healthy and prediabetic adults showed significant reductions in postprandial glucose (up to − 20%) and insulin area under the curve (AUC) (− 10 to − 25%) after 4 to 12 weeks of supplementation [14–17].

Acute trials with RS4 also demonstrated attenuated glycemic responses and increased satiety shortly after consumption [18–20]. In patients with type 2 diabetes, Gargari et al. [21] and Costa et al. [14] reported reductions in fasting glucose and HbA1c following RS2 and RS3 intake, respectively.

On the other hand, some short-duration studies or those using lower doses did not observe significant changes, suggesting a dose-, duration-, and RS-type-dependent effect [22, 23].

### Effects on Lipid Profile and Lipoprotein Metabolism

Findings regarding lipid profiles were positive in about half of the studies. Ni et al. [24] and Meng et al. [25] observed significant reductions in total cholesterol (− 9%) and LDL-c (− 11%) in individuals with NAFLD or early diabetic nephropathy after 8–12 weeks of RS2 (30–40 g/day).

Trials with RS3 reported reductions in triglycerides and modest increases in HDL-c [14, 26], while RS4 studies indicated decreased postprandial TG and increased satiety [19, 27].

Interventions with RS-rich foods such as extruded sorghum [28], resistant maltodextrin [29], and retrograded rice [30] also showed improvements in TG and cholesterol, although to a lesser extent.

### Effects on Blood Pressure and Body Composition

Favorable, albeit modest, effects were observed on blood pressure and anthropometry. Meng et al. [25] and Ni et al. [24] reported average reductions of 3–5 mmHg in systolic blood pressure, associated with improved insulin sensitivity and endothelial function.

Regarding body composition, studies with RS3 [14, 26] and RS2 [31, 32] showed small but significant reductions in body weight and central adiposity. RS4 consumption was also associated with reduced appetite and postprandial glycemia, suggesting an indirect effect on body weight [20].

Other studies did not observe changes, possibly due to lack of caloric control [22, 33]. In contrast, Mohan et al. [34] demonstrated that consuming rice with a higher RS content led to lower postprandial glycemic and lipemic responses, thereby reinforcing the preventive role of RS-rich grains.

## Discussion

### Proposed Physiological and Metabolic Mechanisms

#### Modulation of Glycemia and Insulin Resistance

Resistant starch has been widely investigated for its potential to improve glycemic control and insulin sensitivity through mechanisms including delayed carbohydrate digestion, fermentation by the gut microbiota, and the production of SCFAs. These mechanisms may contribute to reductions in postprandial glycemia and improvements in insulin sensitivity, particularly in individuals with metabolic disorders.

For clarity, the available evidence is discussed according to study design, distinguishing between acute postprandial interventions and longer-term supplementation studies.

##### Acute Postprandial Studies

Several acute clinical trials have evaluated the immediate metabolic effects of RS consumption on postprandial glucose and insulin responses.

Stewart and Zimmer [18] conducted an acute crossover study in healthy adults, evaluating a meal containing RS4, and reported reductions of up to 30% in glucose and insulin area under the curve (AUC) compared with control meals. Similarly, Rahat-Rozenbloom et al. [23] investigated the acute metabolic effects of a meal containing RS2 derived from high-amylose maize starch in healthy adults. They observed significant reductions in postprandial glucose and insulin responses.

Nilsson et al. [35] examined the acute effects of RS intake in healthy individuals, demonstrating that meals containing RS2 increased the secretion of glucagon-like peptide-1 (GLP-1) and peptide YY (PYY) by approximately 15–25%, which was associated with lower postprandial glycemic responses and greater satiety. Comparable findings were reported by Maziarz et al. [32], who evaluated RS2 consumption in healthy adults and observed enhanced incretin responses and improved postprandial glycemic regulation.

These acute findings support the physiological model in which RS reduces the rate of starch digestion in the small intestine while promoting fermentation in the colon, leading to increased SCFAs production and hormonal responses that modulate glucose metabolism.

##### Longer-Term Interventions

Several longer-term clinical trials have evaluated the effects of regular RS consumption on glycemic control and insulin resistance.

Dainty et al. [15] conducted a dietary intervention in adults with cardiometabolic risk, administering 25 g/day of RS2 for 8 weeks, and reported significant improvements in insulin sensitivity, measured by reductions in the HOMA-IR. Similarly, Gargari et al. [21] evaluated patients with type 2 diabetes mellitus who consumed 10 g/day of RS2 for 8 weeks and observed reductions in fasting glucose and glycated hemoglobin (HbA1c).

In individuals with prediabetes, Costa et al. [14] investigated the effects of RS supplementation and reported improvements in glycemic control parameters, reinforcing the potential role of RS as an adjunct dietary strategy for individuals with impaired glucose metabolism.

Additional evidence was reported by Emilien et al. [20], who investigated the effects of 30 g/day of RS4 during a controlled dietary intervention in adults with overweight or metabolic risk. The study demonstrated reductions in postprandial glycemia and carbohydrate cravings, suggesting that RS4 may influence both metabolic and behavioral determinants of glycemic regulation.

Collectively, longer-term interventions lasting 8 to 12 weeks, typically using RS2 derived from high-amylose maize starch, with doses ranging from 15 to 45 g/day, have reported improvements of up to 15% in HOMA-IR and reductions of approximately 10–20% in postprandial glucose levels.

Despite these promising findings, results are not entirely consistent across studies. For example, Zhang et al. [36] observed significant metabolic improvements in healthy adults, whereas Peterson et al. [22], studying individuals with prediabetes, reported no significant changes in glycemic markers. These discrepancies may be explained by differences in baseline metabolic status, RS dosage, exposure duration, and interindividual variability in gut microbiota composition, which can influence fermentation efficiency and SCFA production.

Furthermore, most trials included small sample sizes (≤ 40 participants) and relatively short intervention durations, which may limit statistical power and the ability to detect metabolic changes.

##### Synthesis of Evidence

Overall, current evidence suggests that RS consumption can improve glycemic regulation through multiple pathways, including reduced enzymatic starch digestion, enhanced incretin secretion, and increased SCFA production following colonic fermentation. The metabolic benefits appear to be more pronounced with RS2 and RS4 supplementation, daily doses ≥ 15 g, and intervention periods of at least 8 weeks, particularly among individuals with impaired glucose metabolism.

Nevertheless, substantial heterogeneity persists across RS type, dosage, intervention duration, and population characteristics, underscoring the need for well-controlled, standardized clinical trials to clarify the role of resistant starch in glycemic regulation and cardiometabolic risk reduction.

#### Effects on Lipid Metabolism

The effects of resistant starch (RS) on lipid profile have been investigated in several clinical and dietary intervention studies. However, the magnitude and consistency of these effects vary depending on the RS type, the administered dose, the intervention duration, and the metabolic characteristics of the population studied. Overall, evidence suggests that RS may exert beneficial effects on lipid metabolism, particularly in individuals with pre-existing metabolic disorders.

Some of the most pronounced lipid improvements have been reported in populations with metabolic disease. For example, Ni et al. [24] demonstrated that 12 weeks of RS2 (40 g/day) in patients with NAFLD resulted in a 15% reduction in hepatic triglyceride content, accompanied by improvements in circulating lipid parameters. Similarly, Meng et al. [25] reported reductions in total cholesterol (− 9%) and LDL-c (− 11%) following 12 weeks of RS2 intake in patients with early diabetic nephropathy. The convergence of findings from these two studies suggests that RS2 may exert lipid-lowering effects, particularly in populations with impaired metabolic regulation, potentially by increasing SCFA production during colonic fermentation, which is known to inhibit hepatic lipogenesis and stimulate fatty acid oxidation [37].

Comparable, although generally more modest, improvements have also been observed in individuals without severe metabolic disease. For instance, Johnstone et al. [26] reported a 6% reduction in triglycerides and a 4% increase in HDL-c after 4 weeks of RS3 supplementation (22 g/day) in individuals with overweight, indicating that RS3 may also influence lipid metabolism in populations with moderate metabolic risk. In a similar context, Costa et al. [14] observed reductions in triglycerides and LDL-c following daily consumption of 4.5 g/day of RS3 derived from green banana biomass, suggesting that even relatively lower RS doses may contribute to lipid improvements when incorporated into habitual diets.

Food-based interventions provide additional insight into how the food matrix and RS source may influence lipid outcomes. Mohan et al. [34] investigated a rice variety with high RS content (RS1 + RS3) and reported a 20% reduction in glycemic index and a lower postprandial lipemic peak, suggesting that whole-food sources of RS may simultaneously modulate glycemic and lipid metabolism. Similarly, Lúcio et al. [28] and Astina et al. [29] demonstrated that consumption of extruded sorghum and resistant maltodextrin, both containing significant RS fractions, reduced triglycerides and inflammatory markers, reinforcing the hypothesis that RS-rich foods may exert cardiometabolic benefits through combined metabolic and anti-inflammatory mechanisms.

In addition, Sonia et al. [30] showed that cooling cooked white rice, a process known to increase the formation of RS3 through starch retrogradation, resulted in a lower postprandial glycemic response and modest reductions in postprandial lipemic responses, highlighting how food processing and preparation methods may influence resistant starch formation and subsequent metabolic outcomes.

Additional mechanistic evidence emerges from studies evaluating postprandial metabolism and gut microbial responses. Stewart and Zimmer [27] and Stewart et al. [19] reported reductions of approximately 7–10% in postprandial triglycerides after consumption of RS4, accompanied by increased satiety responses. Complementing these findings, Gentile et al. [31] and Maziarz et al. [32] demonstrated increased fat oxidation and enhanced satiety following RS2 intake, while Ren et al. [38] reported beneficial changes in gut microbiota composition, including increased *Bifidobacterium* and *Roseburia*, together with reductions in systemic inflammatory markers. These findings collectively suggest that part of the lipid-modulating effects of RS may be mediated through a microbiota–metabolism–lipid axis.

Despite these promising results, the literature also shows variability across studies. Differences in RS type (RS2, RS3, RS4), dosage levels (ranging from approximately 4.5 g/day to 40 g/day), intervention duration (acute responses vs. interventions lasting several weeks), and participants’ baseline metabolic status likely contribute to the heterogeneity of findings. For example, larger lipid improvements are observed in studies with higher RS doses and longer intervention periods, particularly in individuals with metabolic disorders. In contrast, shorter trials or those involving metabolically healthy individuals often report more modest or neutral effects.

Taken together, the available evidence suggests moderately consistent improvements in lipid metabolism associated with RS consumption, particularly when RS2 or RS3 are consumed at doses above approximately 20 g/day for at least 4–12 weeks in populations with metabolic risk. However, the variability in intervention protocols and study populations indicates that further well-designed clinical trials are needed to define better the optimal RS type, dose, and duration required to achieve clinically meaningful lipid improvements.

#### Influence on Blood Pressure and Body Composition

Fewer studies have investigated the influence of resistant starch on blood pressure and body composition than on glycemic and lipid outcomes. However, existing evidence suggests that RS may modestly improve cardiovascular and anthropometric parameters. However, results across studies remain somewhat heterogeneous, likely reflecting differences in intervention duration, RS dose, and participants’ baseline metabolic status.

Evidence regarding blood pressure primarily comes from studies conducted in metabolically compromised populations. For instance, Ni et al. [24] reported a reduction of approximately 4 mmHg in systolic blood pressure (SBP) following 12 weeks of RS2 supplementation (40 g/day) in patients with diabetic nephropathy. The consistency of these findings across two independent clinical populations suggests that RS2 may exert beneficial vascular effects, possibly mediated by increased production of SCFAs, particularly propionate, which has been shown to interact with G-protein-coupled receptors (GPR41/GPR43), involved in blood pressure regulation and vascular [39].

Regarding body composition, several studies have reported modest but meaningful improvements in anthropometric measures. Johnstone et al. [26] observed a mean reduction of approximately 1.2 kg in body weight and a decrease of 0.8 cm in waist circumference after 4 weeks of RS3 supplementation (22 g/day) in individuals with overweight. In a similar intervention context, Costa et al. [14] reported reductions in BMI and body fat percentage following daily consumption of 4.5 g/day of RS3 derived from green banana biomass, suggesting that RS-containing foods may improve body composition when incorporated into habitual diets.

Complementary findings were reported by Lúcio et al. [28], who demonstrated improvements in anthropometric indicators and intestinal health parameters following consumption of extruded sorghum enriched with resistant starch, highlighting the potential synergistic role of RS when consumed as part of whole-grain foods containing additional bioactive compounds.

Acute metabolic studies provide mechanistic insights into these changes in body composition. Gentile et al. [31] and Maziarz et al. [32] reported increased fat oxidation and enhanced satiety following meals containing RS2, suggesting that RS may influence body weight regulation by altering energy substrate utilization and appetite control. Similarly, Emilien et al. [20] observed reduced hunger sensations and lower postprandial glycemia after consumption of RS4-enriched products, reinforcing the potential role of RS in modulating appetite-related metabolic responses.

Nevertheless, not all studies demonstrated significant changes in body composition. For example, Peterson et al. [22] and O’Connor and Campbell [33] did not observe meaningful differences in anthropometric outcomes following RS. These discrepancies may reflect several methodological differences across studies, including shorter intervention durations, lower RS doses, the absence of concurrent dietary energy restriction, or differences in participants’ baseline metabolic characteristics. Observational findings from Mohan et al. [34] further suggest that diets dominated by low-RS refined rice may be associated with higher glycemic index and increased risk of weight gain, indirectly supporting the potential benefits of substituting refined carbohydrate sources with RS-rich foods.

Overall, the available evidence suggests that RS consumption may contribute to modest improvements in blood pressure and body composition, particularly in individuals with metabolic risk factors and when consumed regularly over several weeks. However, the evidence remains limited, given the relatively few long-term randomized trials and the variability in intervention protocols. Future studies employing standardized RS doses, longer intervention periods, and precise body composition assessment methods (e.g., DEXA or bioimpedance) will be important to clarify the magnitude and clinical relevance of these effects.

### Limitations and Future Perspectives

Despite promising results, current literature identifies substantial methodological heterogeneity among clinical trials evaluating resistant starch interventions. Therefore, future studies would benefit from more standardized experimental designs. In particular, greater consistency is needed regarding the type of resistant starch used (e.g., RS2, RS3, or other forms), the dose administered, the duration of the intervention, and the food matrix through which RS is provided. In addition, variability in participant characteristics, including baseline metabolic status, body mass index, and the presence of cardiometabolic disorders, may influence metabolic responses and complicate comparisons between studies.

Standardization is also needed in the selection of metabolic endpoints and analytical methods, such as the assessment of postprandial glycemic responses, insulin sensitivity indices (e.g., HOMA-IR), lipid profile parameters, and gut microbiota analyses. Establishing more uniform protocols would improve comparability across studies and clarify the magnitude and consistency of the cardiometabolic effects of resistant starch.

Beyond methodological heterogeneity, there is a notable scarcity of clinical trials investigating the effects of RS1 and RS5. This limitation stems largely from technical and practical factors. RS1, naturally present in intact grains and seeds, has highly variable content depending on processing level, cell structure, and chewing conditions, making it difficult to standardize the dose and accurately quantify RS intake [40]. RS5, formed by amylose–lipid complexes during thermal food processing, remains limited in availability in food-grade form and lacks standardized formulations for clinical research [41]. In contrast, RS2, RS3, and RS4 are preferentially studied due to their greater stability, ease of incorporation into food products, and commercial availability [42]. Although this methodological preference is justified, it restricts a comprehensive understanding of the physiological effects of other RS types, representing a significant gap in the current literature on RS-based nutritional interventions.

Another important limitation identified in the current literature is that future studies would benefit from more standardized experimental designs. In particular, greater consistency is needed regarding the type of resistant starch used (e.g., RS2, RS3, or other forms), the dose administered, the duration of the intervention, and the food matrix through which RS is delivered. In addition, variability in participant characteristics, including baseline metabolic status, body mass index, and presence of cardiometabolic disorders, may influence metabolic responses and complicate comparisons between studies.

Standardization is also needed in the selection of metabolic endpoints and analytical methods, such as the assessment of postprandial glycemic responses, insulin sensitivity indices (e.g., HOMA-IR), lipid profile parameters, and gut microbiota analyses. Establishing more uniform protocols would improve comparability across studies and help clarify the magnitude and consistency of the cardiometabolic effects associated with resistant starch consumption.

## Conclusion

The available evidence suggests that resistant starch (RS) may play a significant role as a functional dietary component in modulating cardiometabolic risk factors. Across the studies analyzed, RS2 and RS3 appear to be the most consistently associated with beneficial metabolic effects, particularly regarding improvements in postprandial glycemic responses, insulin sensitivity, and certain lipid parameters. These effects are frequently attributed to increased colonic fermentation and subsequent production of SCFAs, which may influence glucose metabolism, lipid oxidation, and inflammatory pathways.

In contrast, evidence regarding other forms of resistant starch, particularly RS4, remains more heterogeneous. While some studies report improvements in postprandial triglycerides and satiety responses, results across trials are less consistent, possibly due to differences in chemical modification processes, food matrices, and study designs. Data on RS1 and RS5 in clinical trials remain comparatively limited, preventing definitive conclusions regarding their cardiometabolic effects.

Regarding dosage, the studies included in this review evaluated RS intake ranging approximately from 4.5 g/day to 40 g/day, with the most consistent metabolic improvements generally observed in interventions providing 20 g/day or more of resistant starch over periods of at least 4–12 weeks, particularly among individuals with metabolic disturbances such as obesity, prediabetes, type 2 diabetes, or NAFLD. Lower doses may still produce modest metabolic benefits when consumed regularly as part of whole foods naturally rich in resistant starch.

From a practical dietary perspective, the findings summarized in this review may serve as an initial framework for developing nutritional and dietotherapeutic strategies involving resistant starch. Although the current evidence base remains heterogeneous, the consistent metabolic improvements reported across several clinical studies suggest that increasing intake of foods naturally rich in resistant starch may represent a promising complementary approach to improve cardiometabolic health. In this context, incorporating foods such as vegetables, whole grains, minimally processed cereals, and traditional preparations that favor the formation of retrograded starch—such as cooked and cooled rice, potatoes, and products derived from green bananas—may be considered as preliminary dietary strategies aimed at modulating glycemic responses and metabolic risk factors.

At the same time, the evidence synthesized in this review should be interpreted as a starting point for the gradual development of evidence-based dietary recommendations, rather than as definitive clinical guidelines. Future research involving larger, more representative populations, longer intervention periods, and well-characterized sources of resistant starch will be essential to refine these recommendations and better define optimal intake levels and target populations. Such approaches will help improve the generalizability of findings and strengthen the evidence base for dietary recommendations involving resistant starch.

## Data Availability

All data generated or analyzed during this study are included in this published article.
